# Real-Time Smartphone Guidance Improves Cardiopulmonary Resuscitation (CPR) Performance in Trained and Untrained Individuals: A Stratified Simulation Study

**DOI:** 10.7759/cureus.92196

**Published:** 2025-09-13

**Authors:** Lydia Vallianatou, Theodoros Kapadohos, Maria Polikandrioti, Evangelia Sigala, Evangelia Stamatopoulou, Eleni-Marina Kostaki, Pavlos Stamos, Antonia Kalogianni

**Affiliations:** 1 Nursing, University of West Attica, Athens, GRC; 2 Nursing Education Office, Evangelismos General Hospital, Athens, GRC; 3 Catheterization Laboratory, University Hospital of Athens "Attikon", Athens, GRC; 4 Occupational Therapy, University of West Attica, Athens, GRC; 5 Computer Science, Hellenic American University, Athens, GRC

**Keywords:** bls, ohca, real-time cpr guidance, simulation design, smartphone application

## Abstract

Introduction: Sudden cardiac arrest continues to be a significant cause of death globally, highlighting the importance of performing effective cardiopulmonary resuscitation (CPR). Technological advancements, such as smartphone applications (apps), offer new opportunities to enhance CPR performance.

Purpose: This study aimed to determine whether a specifically designed smartphone app could improve the effectiveness of CPR among trained and untrained individuals in Greece, potentially contributing to better outcomes for out-of-hospital cardiac arrest (OHCA) victims.

Methods: A stratified randomized controlled trial was conducted with 204 adult participants, stratified by Basic Life Support (BLS) certification and randomized to either receive real-time guidance via a smartphone app or not. All participants managed a standardized OHCA simulation using a Quality Cardiopulmonary Resuscitation (QCPR)-enabled manikin (Laerdal Medical, Stavanger, Norway).

Results: Participants using the app demonstrated significantly higher total performance scores and step success rates compared to those without the app (p < 0.001). The effect was particularly pronounced among individuals without prior BLS certification, who achieved greater improvements in both algorithm adherence and chest compression quality. A significant interaction was observed between app use and BLS status (p = 0.001).

Conclusion: Real-time CPR smartphone guidance significantly improved simulated resuscitation performance in both trained and untrained individuals, with the most substantial effect observed in untrained laypersons. Such apps may help bridge the training gap and promote effective bystander intervention during cardiac arrest emergencies.

## Introduction

Out-of-hospital cardiac arrest (OHCA) is among the top three leading causes of death globally [1,2]. Sudden cardiac death (SCD) accounts for 50% of all cardiovascular deaths, with nearly half of these cases occurring as the first cardiac event [3]. Annual records in the US have shown that the number of SCD cases exceeds 350,000 [4]. In Europe, the most recent results from three-month recordings in the EuReCa TWO study indicated 37,054 cases of OHCA [5]. Early detection, immediate cardiopulmonary resuscitation (CPR), and defibrillation within three to five minutes significantly increase the survival rate to 60% or more, with good neurological outcomes [6,7]. The prevalence of bystander CPR (BCPR) initiation varies by nearly 40% across nations [8]. According to EuReCa ONE, there was significant variation in spontaneous circulation (return of spontaneous circulation (ROSC)) and BCPR rates across 27 countries, ranging from 10% to 50% [1]. One significant barrier is the fear of performing CPR incorrectly and possibly doing more harm than good to the victim [9]. This reluctance to perform resuscitation significantly reduces the chances for OHCA patients to be discharged from the hospital and fully return to their everyday lives [10].

Community awareness campaigns and CPR training are essential measures to address this disadvantage [11]. Increasing the BCPR rate and promoting the widespread use of automated external defibrillators (AEDs) can significantly impact the survival rate and reduce the burden of OHCA [12]. The European Resuscitation Council (ERC) and the American Heart Association (AHA) provide citizens with Basic Life Support (BLS) certifications that have an expiration period of three years [6,13]. While certification is an essential component, not everyone who undergoes CPR training can successfully perform high-quality CPR, and this ability is negatively influenced by older age [14].

In this concept, digital tools are an ideal solution for providing users with immediate guidance, personalized information on the performance of chest compressions (CCs), the location of AEDs, or instructions on how to call Emergency Medical Services (EMS) [12,15]. Smartphone applications (apps) could play a pivotal role in this area [16,17]. Numerous apps are available for free download onto devices. Most apps provide BCPRs with audiovisual guidance on performing high-quality CCs and the steps they need to follow [16,18]. However, their effectiveness varies due to certain limitations, such as the heterogeneity of studies resulting from the use of additional wearable devices or the lack of real-time CPR instructions during cardiac arrest [19-22].

This study aimed to examine the contribution of using a specially developed smartphone app in the cardiac arrest scenarios of OHCA victims. Specifically, we sought to investigate whether an app providing real-time guidance could affect participants' life-saving abilities during simulated cardiac arrest scenarios, initially regardless of their prior BLS training, and then according to their prior BLS training.

## Materials and methods

Study design

**Figure 1:**
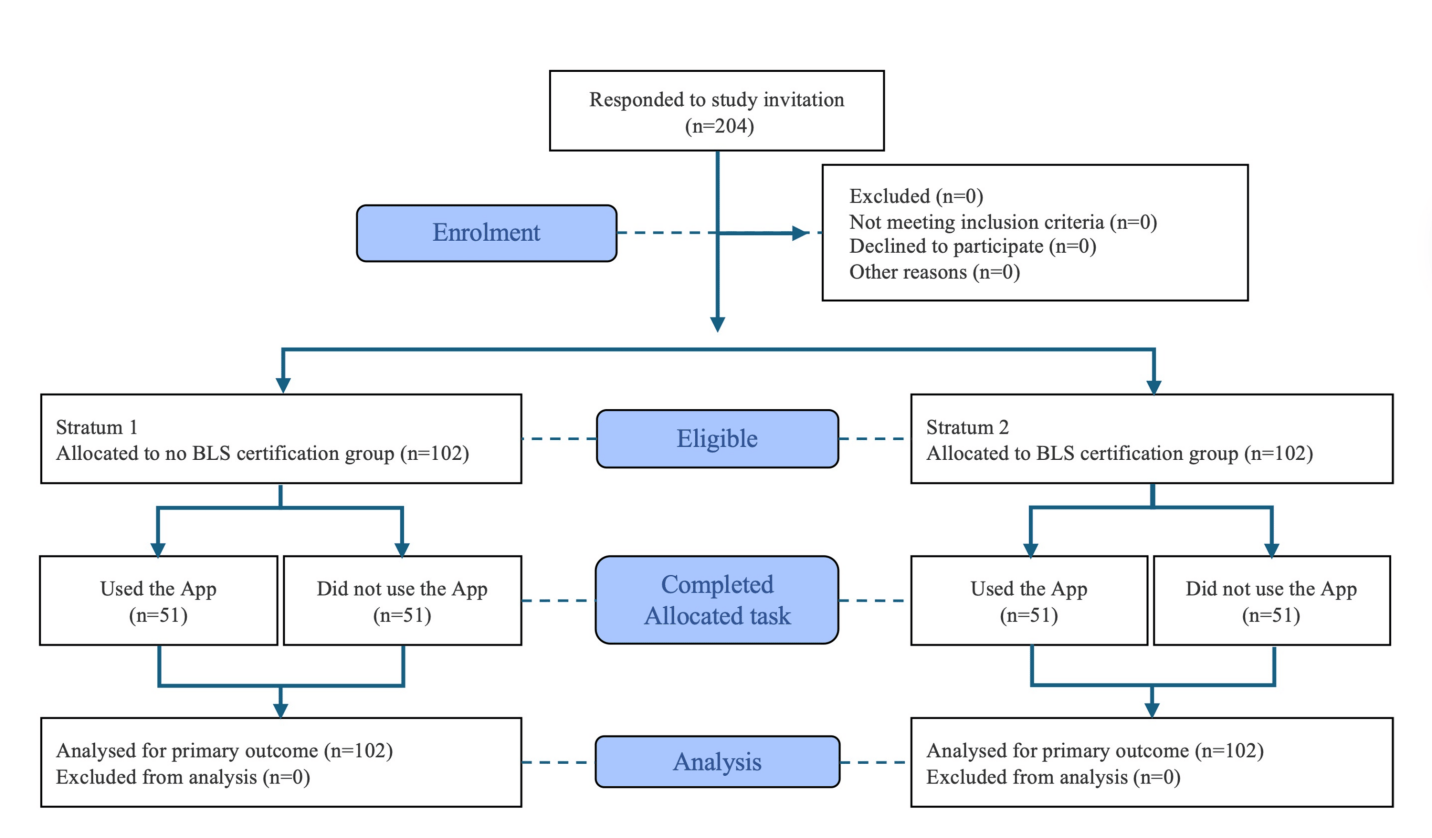
CONSORT flow diagram CONSORT: Consolidated Standards of Reporting Trials; BLS: Basic Life Support

We conducted a stratified randomized controlled trial with a parallel-group design and a 1:1 allocation ratio within strata to evaluate the effect of a smartphone app on adult performance during a simulated OHCA scenario. To ensure a balance between intervention groups, we equally stratified participants based on their BLS certification status, a known prognostic factor influencing CPR performance [23,24]. Within each stratum (with or without prior BLS certification), participants were randomly assigned using a simple lottery method to receive real-time CPR guidance through a smartphone app (App group) or to perform the scenario without such support (Control group). This process resulted in four participant groups (n = 51 per Group): Group 1, no BLS-No App; Group 2, BLS-No App; Group 3, BLS-App; and Group 4, No BLS-App. This effectively fulfilled the purpose of block randomization, as recommended in the Consolidated Standards of Reporting Trials (CONSORT) 2010 guidelines (Figure 1) [25].

**Figure 2:**
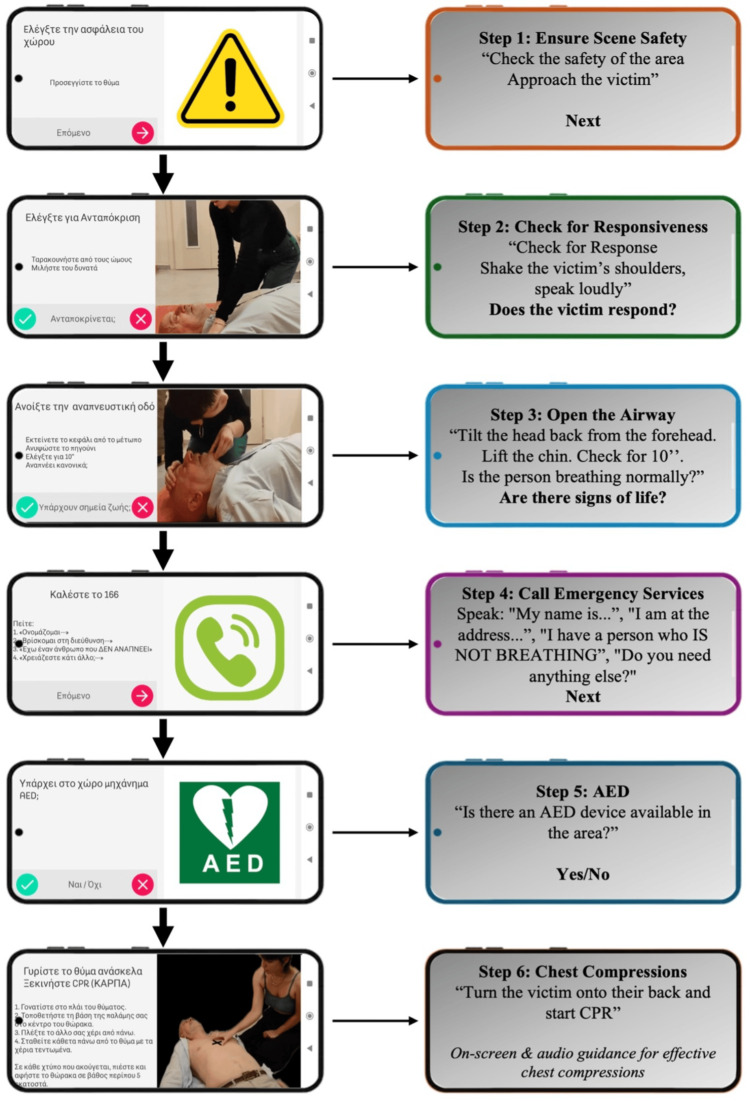
Six steps of the application A series of screenshots from the "Stayin' Alive" app, created exclusively for research purposes to assist users in following the BLS algorithm. The app offers real-time instructions in Greek, complete with video and audio guidance for each step. Alongside the screenshots, English explanatory notes are included. BLS: Basic Life Support; AED: automated external defibrillator; CPR: cardiopulmonary resuscitation

The app design, the used scenario, and the measurements are described in Appendices A-C accordingly. Six steps of the scenario are shown in Figure 2. Criteria for succeeding each step and total score performance calculation are shown in Appendices D, E.

Endpoints

The primary outcome of this study was the effect of the smartphone app on CC quality, as assessed by the total performance score.

Total score calculation: Compression Score = $\dfrac{\text{Depth} \times \text{Rate} \times \text{Chest Release} \times \text{Hand Position} \times \text{Compressions per Cycle}}{\text{Flow fraction}}$

Secondary outcomes included the successful execution of the six steps of the resuscitation algorithm, initiation of CCs, and the qualitative characteristics of CCs measured by the Quality Cardiopulmonary Resuscitation (QCPR)-enabled manikin (Laerdal Medical, Stavanger, Norway) system.

Participants

Participants were recruited from two sources: members of educational institutions and certified lay responders who had completed BLS training through officially recognized institutions at least one year prior to the study.

Included participants were aged between 18 and 67 years, capable of using a smartphone, and, where applicable, certified in BLS within the past one to two years (for the BLS-certified stratum). Individuals who were healthcare professionals (e.g., physicians, nurses, certified EMS providers), pregnant, unable to communicate in Greek adequately, or presented with total or partial physical or mental disability, and BLS instructors were excluded.

Statistical analysis

The distribution of quantitative variables was tested for normality using the Kolmogorov-Smirnov test. For those that were normally distributed, the mean and standard deviation (SD) were used to describe them, while for those that were not normally distributed, the median and interquartile range (IQR) were also used. Absolute (n) and relative frequencies (%) were used to describe categorical and ordinal variables. Pearson’s chi-square test or Fisher’s exact test was used to compare percentages. Student’s t-test was used to compare the age between the app and no-app groups. The non-parametric Mann-Whitney test was used to compare the rest of the quantitative variables between the App and No App groups. The Scheirer-Ray-Hare test was used to test whether the effect of the app on performance scores was associated with the presence of BLS certification. In order to test whether the effect of the app on success rates was associated with the presence of BLS certification, logistic regression analysis was applied, from which the odds ratios (OR) and their 95% confidence intervals (95% CI) emerged. The significance levels are two-sided, and the statistical significance was set at 0.05. Analysis was conducted via IBM SPSS Statistics software, version 27.0 (IBM Corp., Armonk, NY).

Ethical considerations

All participants provided written informed consent prior to their inclusion in the study. Demographic and personal data were ensured to be confidential. A developer created the smartphone app at no cost, specifically for the purposes of this study.

The study received ethical approval from the Research Ethics and Deontology Committee of the University of West Attica, Nursing Department, Athens, Greece (approval code: 48321-19/05/2022). Data collection took place between 2022 and 2023 at the facilities of the Hellenic Cardiology Society (https://www.hcs.gr/en/home/) in Athens, Greece.

## Results

Data from 204 individuals (52% male), with a mean age of 31.9 years (SD=12.2 years), were collected and analyzed. Half of the individuals (n=102) used the app, and the other half did not. The demographics of the participants are presented in Table 1, in the total sample and by group. No significant differences were found between the two groups (p>0.05).

**Table 1:** Sample characteristics in the total sample and by groups The data have been presented as absolute (n) and relative frequencies (%) or as mean (standard deviation) +Pearson’s chi-square test; ++Fisher’s exact test; ‡Student’s t-test; ^1^referred only in those with BLS certificate; App: application; BLS: Basic Life Support; AHA: American Heart Association

Variables		Total sample	No App Group (n=102; 50%)	App Group (n=102; 50%)	χ^2^ value	P-value
		n (%)	n (%)	n (%)		
Gender	Men	106 (52.0)	59 (57.8)	47 (46.1)	2.83	0.093+
	Women	98 (48.0)	43 (42.2)	55 (53.9)		
Educational level	High school	57 (27.9)	33 (32.4)	24 (23.5)	4.56	0.207+
	Two-year post-secondary education	30 (14.7)	12 (11.8)	18 (17.6)		
	University	42 (20.6)	24 (23.5)	18 (17.6)		
	Master's/PhD	75 (36.8)	33 (32.4)	42 (41.2)		
BLS certificate	No	102 (50.0)	51 (50.0)	51 (50.0)	0.00	>0.999+
	Yes	102 (50.0)	51 (50.0)	51 (50.0)		
CPR experience	None	99 (48.5)	50 (49.0)	49 (48.0)	4.35	0.213++
	Internet	3 (1.5)	1 (1.0)	2 (2.0)		
	BLS other	20 (9.8)	6 (5.9)	14 (13.7)		
	BLS/AHA	82 (40.2)	45 (44.1)	37 (36.3)		
Year of certification^1^	2022	60 (58.8)	29 (56.9)	31 (60.8)	0.16	0.687+
	2023	42 (41.2)	22 (43.1)	20 (39.2)		
		Mean (SD)	Mean (SD)	Mean (SD)	t-value	P
Age (years)		31.9 (12.2)	31.6 (11.5)	32.2 (12.9)	0.34	0.732‡

**Figure 3:**
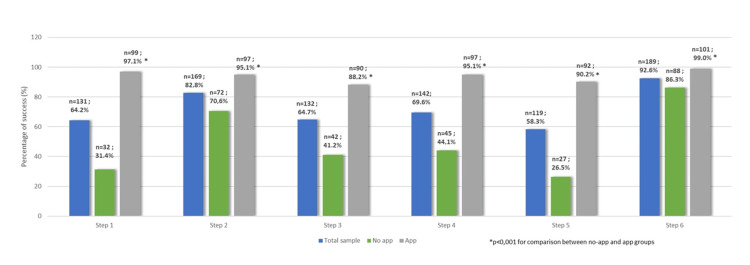
Success rates in the total sample and by group App: application

Participants’ performance scores, in the total sample and by group, are presented in Table 2. Participants who used the app had significantly greater scores in all steps as well as a greater total score (p<0.001). The percentages of participants who succeeded in each step are presented in Figure 3, for all samples and by group. Significantly greater were the percentages of the group that used the app in all steps (p<0.001). Also, among those who succeeded in Step 6, it was found that overall 72% (n=136/189) had placed their hands correctly at the chest. The corresponding percentages of those who did not use the app were 55.7% (n=49/88) and 86.1% (n=87/101) in those who used the app (p<0.001). 

**Table 2:** Participants’ performance scores in the total sample and by group The data have been presented as median (IQR) App: application; AED: automated external defibrillator

Steps	Total sample	No App Group (n=102; 50%)	App Group (n=102; 50%)	U value	P Mann-Whitney test
	Median (IQR)	Median (IQR)	Median (IQR)		
Step 1: Safety	4 (0 ─ 7)	0 (0 ─ 4)	6 (4 ─ 9)	1677.5	<0.001
Step 2: Check for response	5 (3 ─ 8)	4 (0 ─ 6)	7 (4 ─ 8)	2582.0	<0.001
Step 3: Check for breathing	10 (0 ─ 14)	0 (0 ─ 10)	12 (9 ─ 18)	2469.5	<0.001
Step 4: Call ambulance services	9 (0 ─ 13)	0 (0 ─ 8)	12 (9 ─ 17)	1831.0	<0.001
Step 5: AED	2 (0 ─ 3)	0 (0 ─ 2)	3 (2 ─ 4)	2341.5	<0.001
Step 6: Chest compressions start	11 (5 ─ 20)	6 (2 ─ 10.5)	18.5 (11 ─ 22)	1925.5	<0.001
Total score	45 (9 ─ 79)	20 (0 ─ 59)	71 (27 ─ 90)	2654.0	<0.001

The results regarding CCs are presented in Table 3, in the total sample and according to whether or not they had used the app. The total number of CCs, the compression rate, the chest release rate, and the mean depth were similar in both groups (p>0.05). On the contrary, the percentage of compression (p=0.044), the compression time (p=0.021), and the CC fraction (p<0.001) were significantly greater in the App group.

**Table 3:** Participants’ results regarding chest compressions in the total sample and by group *^1^*The data have been presented as median (IQR); *^2^*In this analysis, only participants who succeeded in Step 6 are included. App: application

	Total sample	No App Group(n=102; 50%)	App Group (n=102; 50%)	U-value	P Mann-Whitney test
	Median (IQR)	Median (IQR)	Median (IQR)		
Total chest compression (2 min)	212 (180 ─ 222)	203.5 (154 ─ 225.5)	214 (193 ─ 221)	3915.0	0.158
Compression Average Rate	110 (100 ─ 117)	109 (90 ─ 126.5)	110 (106 ─ 114)	4162.5	0.452
Chest release (%)	99 (69 ─ 100)	99 (75.5 ─ 100)	98 (54 ─ 100)	4235.5	0.561
Compression (%)	86 (7 ─ 99)	68 (1.5 ─ 98)	90 (12 ─ 99)	3694.0	0.044
Compression time	118 (108 ─ 120)	116 (103.5 ─ 120)	119 (112 ─ 120)	3618.0	0.021
Μean depth	2.2 (1.6 ─ 2.6)	2 (1.6 ─ 2.6)	2.3 (1.8 ─ 2.6)	3819.5	0.095
Chest compression fraction	99 (94 ─ 100)	97 (86.5 ─ 100)	100 (97 ─ 100)	3214.5	<0.001

Participants’ performance scores, by group and BLS certification, are presented in Table 4. In participants without BLS certification, the performance scores were significantly greater when using the app (p<0.05). In people with BLS certification, the scores for Steps 1, 3, 4, 5, and 6 were significantly greater when using the app, while for Step 2, the use of the app was not found to significantly alter the scores. Also, the total score was significantly higher in people with BLS certification who used the app compared to people with BLS certification who did not use the app (p=0.003). Comparing participants with and without BLS certification, it was found that, in the No App group, almost all performance scores (except for those in Steps 4 and 6) were significantly greater in the BLS group. Within the App group, the scores in Steps 1, 2, 3, 4, and 6 were significantly lower in the BLS group. Furthermore, It was evaluated whether the app effect differs according to having or not having BLS certification, and it was found that there was a significant difference in the results of Steps 1 (p<0.001), 2 (p<0.001), 3 (p<0.001), and 4 (p<0.001), as well as in the total score (p=0.001). More specifically, it was found that the effect of the app was significantly greater in the aforementioned scores in the no BLS group. Regarding Step 6, the effect of the app on the results of this specific step tended to be greater in the no BLS group (p<0.100).

**Table 4:** Participants’ performance scores by group and BLS certification Note:* *The data have been presented as median (IQR) +U-value and p-value for testing between No App and App groups via Mann-Whitney test; ++ U-value and p-value for testing between having and not having BLS certification via Mann-Whitney test; ‡H-value and p-value for testing if the effect of the app is associated with having BLS certification (via Scheirer-Ray-Hare test) App: application; BLS: Basic Life Support; AED: automated external defibrillator

		No App Group	App Group				
		Median (IQR)	Median (IQR)	U-value+	P+	H value‡	P‡
Step 1: Safety	No BLS	0 (0 ─ 0)	7 (5 ─ 11)	227.5	<0.001	2.78	<0.001
	BLS	2 (0 ─ 6)	5 (3 ─ 8)	714.5	<0.001		
	U-value++	827.5	861.5				
	P++	<0.001	0.003				
Step 2: Check for response	No BLS	0 (0 ─ 4)	8 (5 ─ 10)	373.5	<0.001	4.31	<0.001
	BLS	5 (3 ─ 6)	5 (4 ─ 7)	1039.5	0.078		
	U-value++	738.0	753.5				
	P++	<0.001	<0.001				
Step 3: Check for breathing	No BLS	0 (0 ─ 0)	15 (9 ─ 21)	388.5	<0.001	2.98	<0.001
	BLS	9 (0 ─ 12)	11 (9 ─ 13)	941.0	0.015		
	U-value++	824.0	881.0				
	P++	<0.001	0.005				
Step 4: Call ambulance services	No BLS	0 (0 ─ 6)	15 (11 ─ 20)	332.5	<0.001	1.90	<0.001
	BLS	3 (0 ─ 9)	10 (7 ─ 13)	642.0	<0.001		
	U-value++	1040.0	706.5				
	P++	0.055	<0.001				
Step 5: AED	No BLS	0 (0 ─ 0)	3 (1 ─ 5)	436.5	<0.001	0.46	0.100
	BLS	0 (0 ─ 3)	3 (2 ─ 4)	748.5	<0.001		
	U-value++	956.0	1213.5				
	P++	0.003	0.554				
Step 6: Chest compressions start	No BLS	6 (1 ─ 18)	21 (18 ─ 24)	493.0	<0.001	0.56	0.067
	BLS	6 (3 ─ 9)	12 (9 ─ 20)	412.5	<0.001		
	U-value++	1183.0	631.5				
	P++	0.646	<0.001				
Total score	No BLS	1 (0 ─ 17)	70 (21 ─ 89)	415.5	<0.001	1.76	0.001
	BLS	51 (21 ─ 77)	74 (40 ─ 91)	860.5	0.003		
	U-value++	407.5	1082.0				
	P++	<0.001	0.143				

Regarding the percentages of success, it was found that within the No BLS group, the use of the app resulted in significantly higher success rates in all steps as well as in the correct placement of the hands on the chest (Table 5). In the BLS group, the use of the app resulted in significantly greater success rates in Steps 1, 3, 4, and 5 as well as a significantly higher rate of correct placement of the hands on the chest (n=34; 66.7% vs. n=43; 84.3%, p=0.038). The effect of the app differed significantly depending on whether or not the participants had BLS certification only with regard to Steps 2 and 3, where the difference in success rates of these steps was significantly lower when there was BLS certification (OR=0.07, 95% CI: 0.01 - 0.62, p=0.016 for step 2 and OR=0.15, 95% CI: 0.03 - 0.65, p=0.012 for Step 3).

**Table 5:** Percentages of success by group and BLS certification The data have been presented as absolute (n) and relative frequencies (%). + χ2 value and p-value for testing between no-app and app groups via Pearson’s chi-square or Fisher’s exact test; ‡p-value for interaction term of application and BLS certification (via logarithmic regression), ^1^referred only in those who succeeded Step 6 App: application; BLS: Basic Life Support; AED: automated external defibrillator

			Success				
			n	%	χ^2^ value	P+	P‡
Step 1: Safety	No BLS	No App	6	11.8	69.42	<0.001	0.998
		App	48	94.1			
	BLS	No App	26	51.0	33.12	<0.001	
		App	51	100.0			
Step 2: Check for response	No BLS	No App	26	51.0	26.65	<0.001	0.016
		App	49	96.1			
	BLS	No App	46	90.2	0.54	0.715	
		App	48	94.1			
Step 3: Check for breathing	No BLS	No App	10	19.6	48.34	<0.001	0.012
		App	45	88.2			
	BLS	No App	32	62.7	8.96	0.003	
		App	45	88.2			
Step 4: Call ambulance services	No BLS	No App	17	33.3	43.96	<0.001	0.197
		App	49	96.1			
	BLS	No App	28	54.9	20.65	<0.001	
		App	48	94.1			
Step 5: AED	No BLS	No App	6	11.8	53.77	<0.001	0.884
		App	43	84.3			
	BLS	No App	21	41.2	35.70	<0.001	
		App	49	96.1			
Step 6: Chest compressions start	No BLS	No App	37	72.5	13.21	<0.001	>0.999
		App	50	98.0			
	BLS	No App	51	100.0	-	-	
		App	51	100.0			
Correct placement of hands on the chest^1^	No BLS	No App	15	40.5	33.81	<0.001	0.059
		App	44	88.0			
	BLS	No App	34	66.7	4.29	0.038	
		App	43	84.3			

Participants’ results regarding CCs, by group and BLS certification, are presented in Table 6. In the no BLS group, total CC (p=0.003), compression average rate (p=0.008), compression time (p<0.001), and CC fraction (p<0.001) were significantly greater in individuals who used the app. The average depth was significantly higher in individuals with BLS certification who had the app compared to individuals with BLS certification who did not have the app (p=0.036). Comparing participants with BLS certification and those without one, it was found that, in the no-app group, total CCs (p=0.037), compression rate (p=0.014), compression time (p=0.002), and CC fraction (p=0.002) were significantly greater in the BLS subgroup. In the app group, compression rate (p=0.028) and average depth (p=0.048) were significantly higher in participants with BLS certification. The effect of the app, according to whether or not the participants had BLS certification, was found to differ significantly regarding total CCs (p=0.008), compression time (p=0.015), and compression average rate (p=0.048). More specifically, it was found that the effect of the app was significantly greater in the aforementioned factors in the No BLS group. Regarding CC fraction, the effect of the app on the results of this specific step tended to be greater in the No BLS group (p<0.100).

**Table 6:** Participants’ results regarding chest compressions by group and BLS certification *^1^*The data have been presented as median (IQR); *^2^.* In this analysis, only participants who succeeded in Step 6 are included + U-value and p-value for testing between no-app and app groups via Mann-Whitney test; ++ U-value and p-value for testing between having and not having BLS certification via Mann-Whitney test; ‡p-value for testing if the effect of the application is associated with having BLS certification (via Scheirer-Ray-Hare test) App: application; BLS: Basic Life Support

		No App Group	App Group	U-value+			
		Median (IQR)	Median (IQR)		P+	H value‡	P‡
Total chest compression (2 min)	No BLS	174 (141 ─ 221)	214.5 (200 ─ 221)	581.0	0.003	1.70	0.008
	BLS	210 (170 ─ 232)	212 (182 ─ 220)	1200.5	0.503		
	U-value++	696.5	1057.5				
	P++	0.037	0.139				
Compression average rate	No BLS	100 (88 ─ 112)	110 (108 ─ 112)	617.5	0.008	0.96	0.048
	BLS	111 (97 ─ 127)	110 (100 ─ 117)	1167.0	0.371		
	U-value++	744.0	1251.0				
	P++	0.092	0.87				
Chest release (%)	No BLS	98 (80 ─ 100)	99 (69 ─ 100)	852.0	0.515	0.46	0.164
	BLS	100 (69 ─ 100)	98 (48 ─ 100)	1108.5	0.175		
	U-value++	842.0	1120.5				
	P++	0.361	0.277				
Compression (%)	No BLS	35 (0 ─ 94)	78.5 (0 ─ 99)	740.0	0.105	0.00	0.893
	BLS	77 (31 ─ 99)	96 (68 ─ 100)	1033.0	0.072		
	U-value++	655.5	955.0				
	P++	0.014	0.028				
Compression time	No BLS	110 (94 ─ 119)	119 (113 ─ 120)	543.0	<0.001	1.31	0.015
	BLS	120 (110 ─ 120)	120 (110 ─ 120)	1264.0	0.792		
	U-value++	580.5	1270.5				
	P++	0.002	0.974				
Μean depth	No BLS	2 (1.3 ─ 2.6)	1.95 (1.4 ─ 2.6)	798.5	0.277	0.04	0.694
	BLS	2.1 (1.8 ─ 2.6)	2.4 (2.1 ─ 2.8)	988.0	0.036		
	U-value++	743.5	985.0				
	P++	0.09	0.048				
Chest compression fraction	No BLS	92 (76 ─ 99)	99 (97 ─ 100)	505.5	<0.001	0.74	0.058
	BLS	99 (94 ─ 100)	100 (97 ─ 100)	1085.0	0.114		
	U-value++	581.0	1162.5				
	P++	0.002	0.404				

## Discussion

This stratified study aimed to investigate the impact of smartphone app use on CPR performance among adults, with or without BLS certification, during a manikin-simulated cardiac arrest scenario. The findings demonstrated that both app use and prior BLS training independently contributed to significantly higher performance scores (p < 0.001 for both factors). Moreover, a significant interaction was identified between app use and BLS certification status (p = 0.001), suggesting that the app was particularly beneficial for participants without prior training. These findings highlight the complementary role of digital tools and structured training in enhancing resuscitation performance, particularly among laypersons.

Implications of real-time CPR guidance through mobile technology

The use of a smartphone app as a tool to guide CPR in real-time is highly effective, has improved CPR performance in trained participants, and has encouraged untrained laypersons to attempt resuscitation [26]. This feature is especially relevant since Greece lacks a system for telephone-assisted CPR, unlike many other countries [27-29]. It is also a valuable tool for guiding an untrained bystander through CPR and helping a trained person recall their training and execute it effectively in high-pressure scenarios [30,31]. An area in which this technology may have a significant impact is in the home, where more than 70% of all OCHA cases occur [32].

It is worth noting that our app gives the instructions through an automated video. Video can enhance self-learning to perform CPR, so in some countries, National EMS services have created an app for video-assisted CPR (v-CPR) [33,34]. In countries such as Denmark [35] and Korea [36], improved first responder performance and better neurological outcomes have been recorded due to live video transmission to the medical dispatcher with real-time feedback. Similarly, real-time guidance provided by technological tools, such as our app, may enhance CPR quality. However, there are few real-life studies, and most countries have not yet developed such systems. Considering that our app provides video guidance, its use could significantly contribute to an emergency event.

CPR smartphone app: a powerful tool to save lives with or without certification?

Our findings confirmed that using the smartphone app, regardless of BLS certification status, significantly improved CPR performance. This finding suggests that combining formal certification with real-time digital support may lead to optimal CPR performance [16,21]. However, it is also evident that skills acquired through training may diminish over time, particularly without ongoing refreshers or technological reinforcement [21]. During the simulation, the app appeared to empower users, supporting them in executing life-saving actions, and in some cases, matching or even surpassing the performance of trained individuals without the app’s support [16]. Beyond guiding step-by-step actions, the app may serve as a motivational aid, helping untrained individuals overcome hesitation and act confidently during emergencies, addressing one of the most frequently cited barriers to bystander CPR: the fear of doing harm [9].

An interesting observation was that a person who was listening to the app instructions was more likely to consistently check the scene and personal safety and give all the necessary information to EMS. Another important finding was that even untrained people attempted CCs, possibly influenced by the study's context or general knowledge through the media. This may reflect the effectiveness of public awareness campaigns and media portrayals of CPR [11,37]. Moreover, the app guided these untrained bystanders through the process of CPR and significantly motivated them to attempt CPR. Additionally, the app was effective in guiding users on the placement of hands for CCs, a factor that significantly influences CPR quality [38]. Our results on the performance of quality attributes of CCs are confirmed in other studies, where both redundancies and limitations were similarly identified [20,25,37-40].

One disadvantage observed in untrained subjects was the significant delay in initiating CPR when using the app, with a median time of 21 seconds to first CC. This delay may be attributed to the length and structure of the narrated or video-based instructions, highlighting the need for an optimized instructional design that maintains clarity while reducing response time. Similar findings have been reported in previous studies, where app use was associated with longer hands-off intervals or delayed compressions [30,37,38].

Limitations

The limitations of this study are mainly due to the use of manikins in the simulation approach, which may not fully represent the reality of CPR practice in the field. Moreover, unlike in real-life scenarios where CPR with the help of a dispatcher is available, the participants were acting independently. The duration of the cardiac arrest simulation (two minutes of CCS) may be shorter than the duration of the real-life simulation [41]. There was a potential bias in selection, as participants knew the study title but not the details of the procedures, the app, or the time of familiarity, minimizing bias. Finally, in the use of a robust feedback program (QCPR), the direct performance reporting can be questionable [26, 36]; this is cross-checked by a visual check. Although chest compression fraction differences were statistically significant, both groups exceeded the >80% threshold, which is generally accepted as high-quality CPR, thus limiting the clinical significance of this primary outcome. Nevertheless, it should be noted that the app markedly increased the proportion of participants who initiated CCs, highlighting its practical relevance.

## Conclusions

This study demonstrates that real-time CPR smartphone apps can significantly enhance the ability of both trained and untrained individuals to initiate and perform high-quality resuscitation during simulated OHCA scenarios. In particular, the app clearly increased the likelihood that untrained participants initiated compressions and followed key BLS steps, underscoring its practical impact. Moreover, it highlights the potential of such tools to evolve further through continued research and iterative design, thereby maximizing usability in diverse emergency contexts. Smartphone apps represent a critical advancement in the prehospital management of emergencies, particularly OHCAs. Future research should focus on optimizing instructional design and evaluating app performance in real-life scenarios to strengthen its role in emergency health responses.

## Appendices

Appendix A

Application

For the purposes of this study, we developed a Greek-language smartphone application called Staying Alive, which was developed solely for research purposes and is not commercially available. There are no plans for its distribution in app stores at this stage, and it will not be monetized. The App delivers real-time audiovisual prompts covering all steps of the BLS sequence, including opening the airway, assessing breathing, and initiating chest compressions. It features automated video demonstrations, voice instructions, and a built-in metronome to help users maintain the correct compression rate throughout the scenario. Participants followed hands-only CPR protocols and did not perform rescue breaths. This decision was based on observations during the pilot testing phase, conducted shortly after the COVID-19 pandemic, where many participants either hesitated or omitted rescue breaths entirely.

Appendix B

Scenario

We presented each participant with the same scenario in which they had to identify a cardiac arrest (CA) victim and apply all steps of the BLS algorithm. The investigators allowed the scenario to proceed without intervening. In the formal study period, only a few participants with BLS certification expressed interest in administering rescue breaths. These participants were the sole exception, receiving guidance from the investigators to perform a hands-only CPR, an approach supported by official organizations for OHCA cases, which encourages bystanders not to hesitate in assisting in public areas [23].

Appendix C

Measurements

Groups using the CPR smartphone application received an introduction describing how to start the application. Before the start of the study, a pilot application was conducted on 5% of the participants to identify potential issues related to the use of the smartphone app. We did not include these pilot participants in the main study analysis. The simulation template used in this study is of the Laerdal company, the "Little Anne QCPR" model. We carried out recordings using the QCPR application.

We designed a six-step protocol to evaluate CPR performance, recording the time taken to complete each step for data analysis purposes. Each step was timed separately using a stopwatch, which we reset to zero at the beginning of each new step. If a participant skipped a step, the recorded time for that step was 0. However, the successful completion of these steps was judged based on the criteria of action completion rather than the time taken, except for Step 3 (Breath Check). For step 3, we defined success as completing the assessment within the recommended time frame of 8 to 30 seconds.

The Laerdal QCPR-enabled device rates CPR performance from 0% to 100%. Compression depth, compression rate, incomplete release, number of compressions per cycle, and hand position are all factors that affect the compression score. The percentage of time that compression was applied, known as the flow fraction, is crucial for evaluating the effectiveness of CPR. It represents the proportion of time that compressions were administered, incorporating elements such as depth, rate, chest release, and hand position into the overall compression score.

Scores are lowered along S-curves outside the thresholds when CPR performance deviates from the recommendations. This deviation implies that minor deviations result in minor score reductions, while more significant deviations will result in substantial score reductions. The Total Score was calculated as follows (Supplementary Appendix 3): Compression Score (depth, frequency, chest release, hand position, compressions per cycle) / flow fraction.

Appendix D

Criteria for Defining Success in CPR Steps During the Study

Steps were deemed failed if not performed at all. In the case of the Breath Check, failure was defined either as not performing the step at all or performing it outside the designated time frame. For participants who managed to initiate CPR (succeeding step 6), we calculated the qualitative characteristics of CCs: the time the victim (manikin) remained without circulation, the total number of CCs, the frequency of CCs in a two-minute CPR cycle, the compression rate, chest release, and the proportion of CCs. The criteria of each step are as follows:

Step 1. Safety: Success was defined as the participant looking for safety. Results showed that almost all the participants using the app successfully made a safety check, thus pointing out the app's potential to enhance safety awareness during emergency situations.

Step 2. Check for Response: Whether the participants checked if the victim was responsive or not; this is an essential initial response to a victim, which the app leads through in detail, even for users untrained in such scenarios, thus commanding high compliance rates.

Step 3. Check for breathing: We defined success as checking breathing within 8 to 30 seconds, driven by these recommended times for a quick assessment. This timing points to the need for further refinement in the instructional pace of the app to allow for a quicker response while maintaining accuracy.

Step 4. Call Emergency Services: Participants were noted to make an emergency call; this was a point where the application reminded the users to notify emergency services, which is the most critical step often forgotten even by trained professionals.

Step 5. AED Request: This step was considered complete if participants requested an Automated External Defibrillator (AED). Importantly, the app users were appropriately reminded of this step, which is often forgotten, especially by those who are unfamiliar with the critical role of AEDs in resuscitation.

Step 6. Start Chest Compressions: In this final step, we counted an attempt to start chest compressions as a success, irrespective of the technique applied. The willingness of untrained individuals to engage, even without app assistance, was remarkable and suggests a growing awareness and readiness to perform CPR.

Appendix E

Total Score Performance Calculation

The variable “total score performance” was calculated automatically by the QCPR application of Laerdal (https://laerdal.com/support/scoring/). According to the application manual, the overall score consists of three components: compression, flow fraction, and ventilation scores. However, when ventilation is not included in the assessment, the total score is calculated using only the compression score and flow fraction score, with adjustments to ensure that the performance assessment remains accurate during hands-only CPR. The Compression Score is calculated from the compression depth, compression rate, incomplete release, number of compressions per cycle, and hand position. The Flow Fraction Score represents the percentage of time during the resuscitation effort that the compressions are actively delivered.
